# Cancer metastasizes to the bone marrow and not to the bone: time for a paradigm shift!

**DOI:** 10.1007/s00259-018-3959-6

**Published:** 2018-02-21

**Authors:** Poul Flemming Høilund-Carlsen, Søren Hess, Thomas J. Werner, Abass Alavi

**Affiliations:** 1 Department of Nuclear Medicine, Odense University Hospital, Odense, Denmark; 2 Department of Clinical Research, Faculty of Health Sciences, University of Southern Denmark, Odense, Denmark; 3 Department of Radiology and Nuclear Medicine, Hospital of Southwest Jutland, Esbjerg, Denmark; 4 Department of Radiology, Hospital of the University of Pennsylvania, Philadelphia, PA 19104 USA

Bone metastases represent a serious complication to several cancers, in particular breast, lung, and prostate cancer. Since their presence influences prognosis and management, they must be searched for. Thus, the introduction of skeletal radionuclide imaging with ^18^F-NaF (NaF) 55 years ago was a major achievement [1], as was its replacement, scintigraphy with bone-seeking phosphates [2], until computed tomography (CT), magnetic resonance imaging (MRI), and once again NaF in the shape of NaF-positron emission tomography/CT (NaF-PET/CT) began contending for precedence. However, this development took place gradually, without anybody asking the central questions: What are skeletal metastases, how do they arise, and how do we best discover them?

**Fig. 1:**
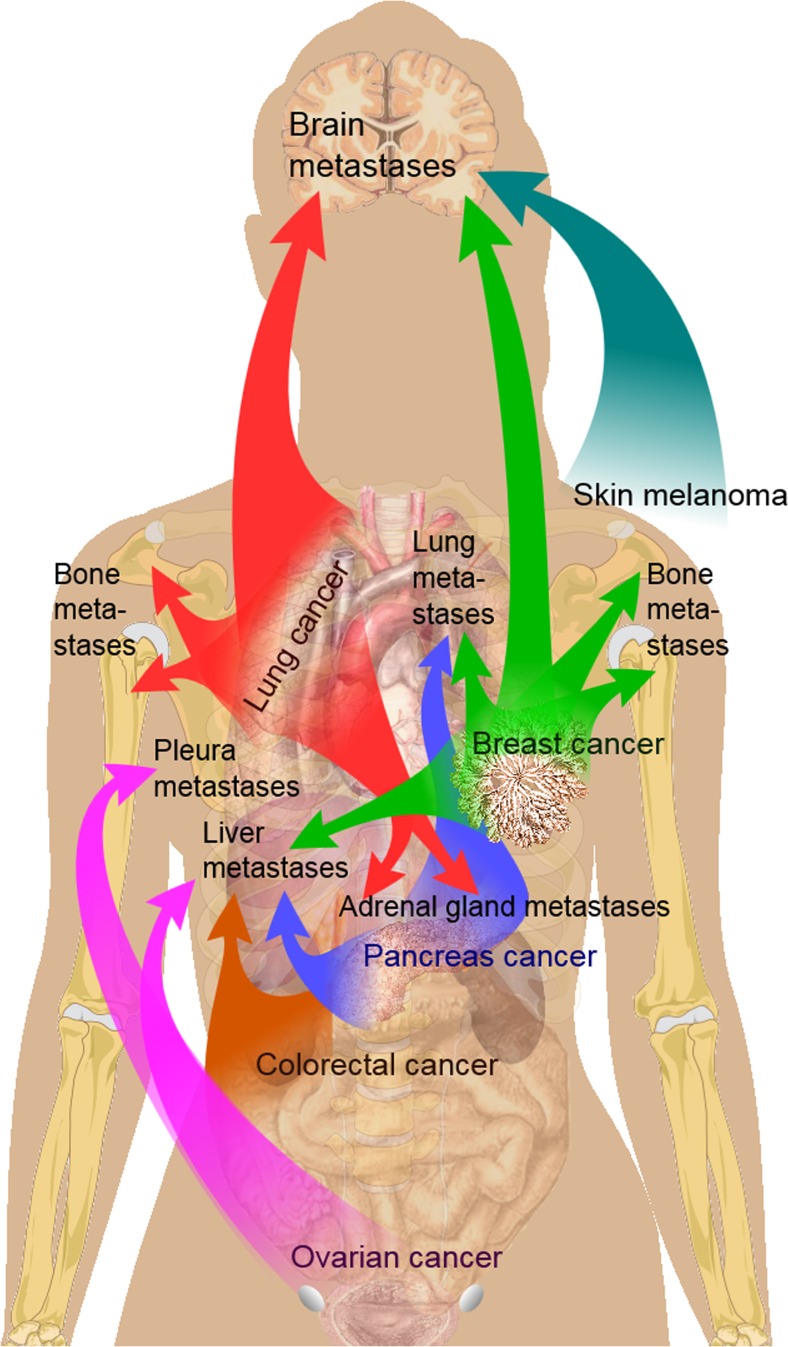
Main sites of metastases for some common cancer types, with lung and breast routes to bones shown at shoulder level. Prostate cancer, the third major source, is not shown because of the female model. Primary cancers are denoted by “*...cancer*,” and their main metastasis sites are denoted by “*...metastases*.” Source: Wikipedia. Bone metastases. The images are in the public domain [3]

A search of the Internet does not yield proper answers. From Wikipedia [3], it appears that:

- “Bone metastases generally arise from epithelial tumors and form a solid mass inside the bone.” “Bone is the third most common location for metastasis, after the lung and liver. While any type of cancer is capable of forming metastatic tumors within bone, the microenvironment of the marrow tends to favor particular types of cancer, including prostate, breast, and lung cancers. Particularly in prostate cancer, bone metastases tend to be the only site of metastasis. The most common sites of bone metastases are the spine, pelvis, ribs, skull, and proximal femur” (Fig. 1) [3].

Wikipedia articles may be changed or edited frequently and thus cannot be taken as peer-reviewed scientific material. If instead one turns to professional websites like those of the Mayo Clinic or the American Cancer Society [4, 5], one can read:

- “Under normal conditions, bone undergoes a continuous remodeling through osteoclast-mediated bone resorption and osteoblast-mediated bone deposition. These processes are normally tightly regulated within bone to maintain bone structure and calcium homeostasis in the body. Dysregulation of these processes by tumor cells leads to either osteoblastic or osteolytic phenotypes, reflective of the underlying mechanism of development. Typically, osteolytic metastases are more aggressive than osteoblastic metastases, which have a slower course. Regardless of the phenotype, though, bone metastases show osteoclast proliferation and hypertrophy” [4].
- “Bone metastasis can occur in any bone but more commonly occurs in the spine, pelvis and thigh. Bone metastasis may be the first sign that you have cancer, or bone metastasis may occur years after cancer treatment” [5].

None of these sites clearly specify what bone metastases are, how they occur, or how best to detect them, even though they mention “medical imaging” and biopsy. The Wikipedia webpage on bone marrow does not once mention bone marrow metastases or bone metastases, and its section on “Imaging” deals exclusively with CT, conventional x-ray, and MRI—not PET [6]. The American Cancer Society *Guidelines for the Early Detection of Cancer* [7] and its guidelines for the early detection of breast, lung, and prostate cancer specify mammography, CT, and MRI, without mentioning bone metastases or PET. The same goes for the 2007 World Health Organization (WHO) guideline for early cancer control [8] and the recent EU publication *Cancer Screening in the European Union* [9]. Oncologists and radiologists often refer to the Response Evaluation Criteria in Solid Tumors (RECIST) guideline, which designates lesion size of at least 10 mm as a criterion for malignancy, which of course does not make sense, and does so without mentioning bone or bone marrow metastases [10]. Hoping to finally learn about how to examine for skeletal metastasis, we took a look at the elaborate Danish National integrated cancer pathways, supported by the Danish government and the five Danish regions [11]. They have since 2009 guided management of all cancer patients in the country, where 95% of healthcare is free and public. However, not one of 29 pathways mention PET as a potential first-line examination in any cancer, not even lung, and we could not find a single one telling how to diagnose skeletal metastases [12].

Amazing to say: none of these official expert consensus documents distinguish between what happens in the bone marrow and bone matrix or describe when and how osseous bone is affected. They list conventional x-ray, CT, and MRI for screening or as first-line diagnostic imaging modalities, saying almost nothing about detection of skeletal metastases. This lack is in stark contrast to the fact that the presence or absence of metastases, including skeletal metastases, is crucial for the choice of treatment, as illustrated by the recent heated debate regarding whether aggressive and more targeted therapy in patients with so-called oligometastatic disease is justified, the rationale being that this may increase life expectancy of selected cancer patients [13–16].

In short, guidelines and recommendations neglect, disregard, or minimize the importance of bone marrow metastases, their pathobiology, and how to best diagnose them at an early time point, when they are supposedly more easily treatable. This is an unfortunate trend in the twenty-first century: to hang on to traditional thinking and conventional indirect and suboptimal techniques that can only detect osseous bone changes when skeletal metastases have established themselves as bone marrow metastases and at some unknown time point have caused structural bone changes. Equally misleading is that the reactive changes that they can detect may persist long after the active cancer cells have disappeared, as a result of efficient chemo- and/or radiotherapy.

Therefore, it is timely to state: *Skeletal metastases are bone marrow and not bone metastases!* The true nature of skeletal metastasis being bone marrow metastasis was highlighted 10 years ago by Basu et al. [17, 18], but apparently was overlooked or ignored, although one should think that their detailed account would suffice to convince even tenacious supporters of the indirect imaging methods, especially if seeing it from the patient’s viewpoint. Skeletal metastases develop as a multistep process. When circulating tumor cells home to and invade the highly vascularized bone marrow as metastatic seeds, there follows a lag phase of unknown length comprising a dormant phase and a more aggressive active phase, both of which are not only regulated and modified by a huge number of factors in the microenvironment of the bone marrow stroma, but are potentially mediated via sympathetic stimulation as well [19–21]. In experimental tumor models, the dormant phase may be relatively short, but in the clinical setting, such periods of latency may last years, at least following excision of primary tumors. Moreover, the tumors developing from metastatic disseminations to the marrow exhibit a range of phenotypes which may be characterized by the relative activity of tumor-associated osteolysis and tumor-induced bone formation [19]. Thus, metastatic processes are present in the bone marrow long before they give rise to the derangement of normal bone tissue architecture, which is what is detectable by conventional x-ray, CT, and SPECT and PET with bone-seeking radiopharmaceuticals. Therefore, these modalities bear only indirect and not real-time evidence of existing active tumor cells in the skeleton, and the changes they depict may persist for long periods even if the cancer cells have been eradicated.

Nonetheless, there are multiple comparisons in the literature of conventional x-ray, CT, and SPECT in bone metastases, and new ones continue to appear, now also with NaF-PET/CT and NaF-PET/MR [22, 23] including a recent article which states that there are no differences between planar bone scintigraphy, SPECT and SPECT/CT, and NaF-PET/CT and NaF-PET/MR in diagnosing bone metastases [24]. However, while such difference may be absent, the message is misleading, because the comparisons included only indirect methods, which depict bone changes and not the all-important bone marrow metastases.

The consequence is that we must start using imaging techniques that can detect, and preferably quantify, bone marrow metastases as early as possible to guide management and increase the possibility of cure, whereas indirect methods should be abandoned. The only methodologies that can offer this today are PET/CT and PET/MRI, applying FDG or some more cancer-specific tracers such as prostate-specific membrane antigen (PSMA)-associated probes. We suggest that detection and quantification of bone marrow metastases should in the future be based on PET/CT and possibly PET/MR imaging, applying tracers that target and depict the degree of malignancy of active cancer cells proliferating in the red bone marrow. For this purpose, FDG-PET is an excellent choice in most cancers because it reflects tumor biology [25], meaning that the rate of FDG uptake is an indication of the aggressiveness of tumor and metastasis and thus a valuable indicator of prognosis. In fact, since cancers may differ in geno- and phenotype from the primary, to regional to distant metastasis including bone marrow metastasis [26], and because bone marrow metastases comprise several phenotypes [19], the criticized lack of specificity of FDG appears to be an advantage.

**Fig. 2:**
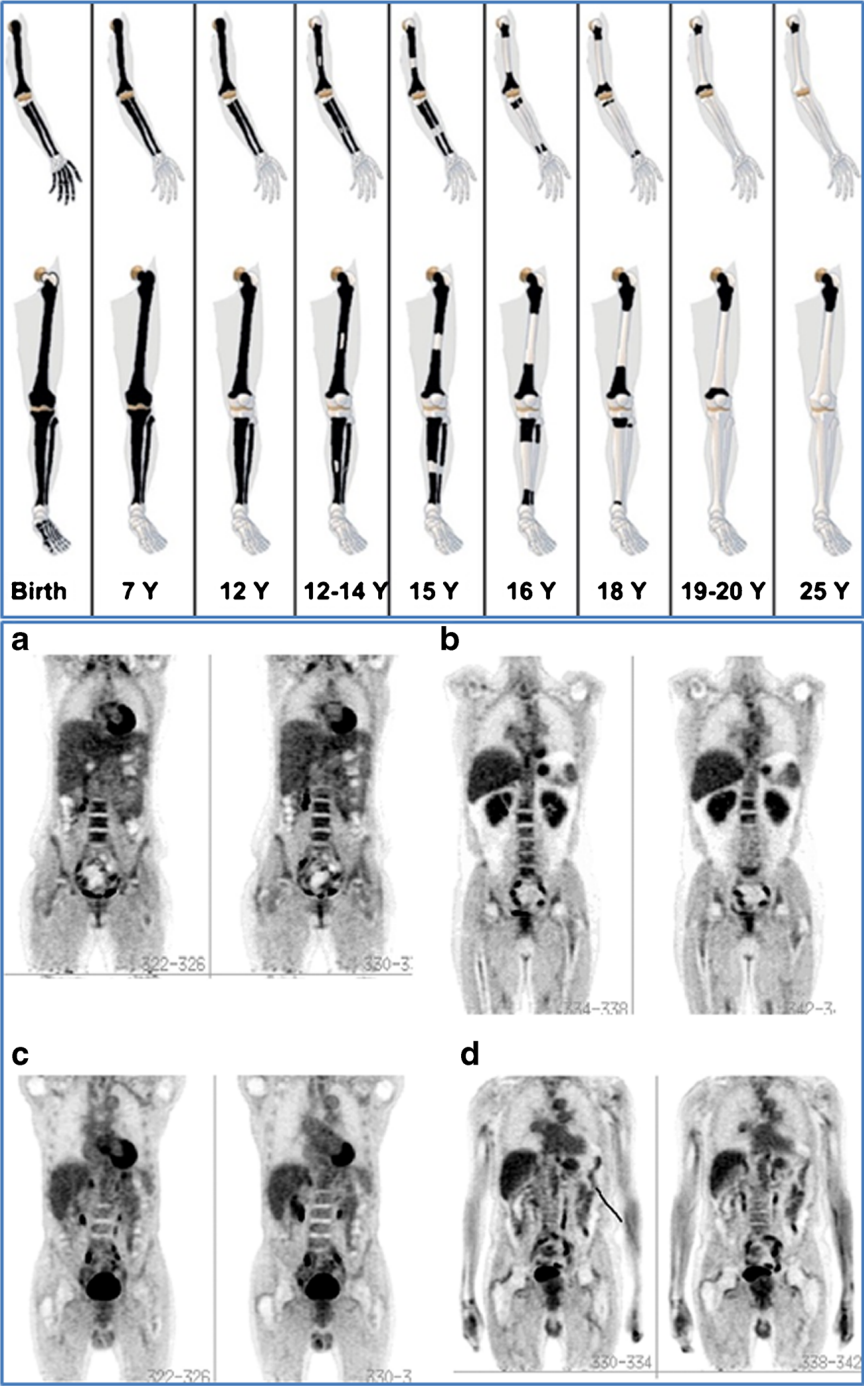
Upper panel: schematic representation of the natural shrinkage in red bone marrow which resides throughout the skeleton in the early years of life and shrinks back to the axial skeleton at the age of 20. This explains why metastases in the extremities are rarely seen in adult patients, and underlines the necessity of imaging the entire skeleton when looking for skeletal metastases in children, whereas stopping at the mid-thigh is defendable in adults. Lower panel: whole-body FDG-PET images of subjects (a) younger than 20 years, and (b) 35, (c) 55, and (d) 75 years of age. Note that red marrow activity and distribution in the spine, pelvic bones, and femora are more prominent at a younger age. Reprinted with permission of reference [27]

When looking for metastases to the skeleton, it is useful to keep in mind that the red bone marrow in the first years of life is present all the way to the distal end of our extremities, and shrinks back to the axial skeleton during childhood (Fig. 2) [27]. Some skeletal metastases from slow-growing tumors such as thyroid, some types of breast, and prostate cancer may be better assessed with more specific agents, the latter with, for instance, PSMA-associated tracers. On the whole, PET with tracers depicting cancer cells, their metabolism or other useful characteristics, and not reactive changes in the surrounding osseous tissue, are preferable—not only because they depict active cancer, but also because these techniques provide otherwise impossible quantification of the extent and severity of cancerous skeletal involvement.

There are few comparisons with the most obvious molecular imaging modality in cancer, FDG-PET/CT. They show that FDG-PET/CT is superior in lung and breast cancer [28–30]. There is no evidence that the combination of FDG and NaF is warranted except in rare cases, because NaF-PET/CT does not detect more osseous lesions than FDG-PET/CT [31]. Moreover, NaF PET/CT has other challenges to deal with. It has high specificity for osteoblastic lesions, but a generally low specificity is a larger problem with NaF than FDG, as many benign lesions light up for a very long period of time on NaF-PET/CT and because degenerative changes, the most frequent abnormalities depicted by this modality, may have intense and long-lasting NaF uptake, while FDG often shows only weak accumulation. Healed fractures are often FDG-negative, but can remain NaF-positive for a long time [31]. The literature lacks prospective longitudinal studies with FDG-PET which show the temporal development of bone marrow to bone metastases, and that the changes seen with CT and SPECT during and after chemotherapy treatment do not necessarily represent cancer any longer. In an elegant small retrospective study, it was recently demonstrated rather convincingly that marrow metastases are the early form of skeletal metastases in breast cancer, and that early systemic treatment may preclude the development of bone metastases [32].

In conclusion, we strongly advise that the concept of bone marrow, instead of bone metastases, replace current misconceptions and current indirect imaging technologies, and bring about much needed changes in the day-to-day practice and guidelines regarding how to detect, gauge, and treat skeletal metastases. Those who still believe in x-rays and bone scintigraphy for this purpose may claim that access to PET is limited and that the procedure is too costly. This has little bearing, as FDG-PET has been shown to be cost-effective in various cancers [33] and will prove to be so in bone marrow metastases as well. We are dealing with a fatal disease of growing incidence which requires accurate and individualized assessment to guide treatment and improve the prospects of affected patients. No method other than PET, used correctly, is as well suited to provide this.
